# Developing Theory-Driven, Evidence-Based Serious Games for Health: Framework Based on Research Community Insights

**DOI:** 10.2196/11565

**Published:** 2019-05-02

**Authors:** Sarah Verschueren, Connor Buffel, Geert Vander Stichele

**Affiliations:** 1 MindBytes BVBA Merksplas Belgium; 2 MindLab Interactive AI Inc Edmonton, AB Canada

**Keywords:** health, computer games, digital, intervention, review, methodology

## Abstract

**Background:**

The idea of using serious games to effectuate better outcomes in health care has gained significant traction among a growing community of researchers, developers, and health care professionals. Many now recognize the importance of creating evidence-based games that are purposefully designed to address physical and mental health challenges faced by end users. To date, no regulatory resources have been established to guide the development of serious games for health (SGH). Developers must therefore look elsewhere for guidance. Although a more robust level of evidence exists in the research literature, it is neither structured nor is there any clear consensus. Developers currently use a variety of approaches and methodologies. The establishment of a well-defined framework that represents the consensus views of the SGH research community would help developers improve the efficiency of internal development processes, as well as chances of success. A consensus framework would also enhance the credibility of SGH and help provide quality evidence of their effectiveness.

**Objective:**

This research aimed to (1) identify and evaluate the requirements, recommendations, and guidelines proposed by the SGH community in the research literature, and; (2) develop a consensus framework to guide developers, designers, researchers, and health care professionals in the development of evidence-based SGH.

**Methods:**

A critical review of the literature was performed in October to November 2018. A 3-step search strategy and a predefined set of inclusion criteria were used to identify relevant articles in PubMed, ScienceDirect, Institute of Electrical and Electronics Engineers Xplore, CiteSeerX, and Google Scholar. A supplemental search of publications from regulatory authorities was conducted to capture their specific requirements. Three researchers independently evaluated the identified articles. The evidence was coded and categorized for analysis.

**Results:**

This review identified 5 categories of high-level requirements and 20 low-level requirements suggested by the SGH community. These advocate a methodological approach that is multidisciplinary, iterative, and participatory. On the basis of the requirements identified, we propose a framework for developing theory-driven, evidence-based SGH. It comprises 5 stages that are informed by various stakeholders. It focuses on building strong scientific and design foundations that guide the creative and technical development. It includes quantitative trials to evaluate whether the SGH achieve the intended outcomes, as well as efforts to disseminate trial findings and follow-up monitoring after the SGH are rolled out for use.

**Conclusions:**

This review resulted in the formulation of a framework for developing theory-driven, evidence-based SGH that represents many of the requirements set out by SGH stakeholders in the literature. It covers all aspects of the development process (scientific, technological, and design) and is transparently described in sufficient detail to allow SGH stakeholders to implement it in a wide variety of projects, irrespective of discipline, health care segments, or focus.

## Introduction

### Background

Many games and apps market themselves as tools or interventions to address health conditions and disease, yet they provide little explanation on their development, provide minimal information on real-world evaluation of their efficacy, and often reference poorly designed research studies [1-4]. Developers, designers, researchers, and health care professionals involved in the development of serious games for health (SGH) (further simply referred to as *developers*) now increasingly recognize the importance of creating evidence-based games that are purposefully designed using expertise, knowledge, and validated, quality data [1-7]. To be recognized as a nonpharmacological health care intervention and gain marketing approval from regulators, to obtain reimbursement from health care payers, or to gain CE approval when dispensing medical advice, developers will need to follow rigorous standards and provide a solid rationale for use and clear empirical evidence of the intervention’s safety and efficacy [8]. This trend, together with an increasing focus on incorporating patient needs and preferences in the development process of health care interventions [9-11], has resulted in a paradigm shift in the development of SGH from a mainly game design orientation with a focus on user experience toward a more scientific approach that involves multiple stakeholders such as patients, clinicians, caregivers, payors, as well as regulators [12,13].

Developers who intend to market their SGH interventions to clinicians and patients will also need to deliver convincing evidence of the game’s ability to safely achieve the intended outcomes if they wish to overcome the current barriers to uptake. As only a few validated tangible success stories exist, many clinicians are skeptical about the use of SGH in current health care practice. These barriers may hinder medical and scientific progress in certain fields and impact the investment risk associated with developing SGH. Although the development cost can vary greatly depending on complexity, graphical and technical design features, and the time spent on scientific substantiation and (clinical) evaluation, it typically ranges from ten to several hundred thousand dollars [14]. When complex three-dimensional motion graphics, community platforms, or large-scale clinical evaluation trials are involved, the development costs can even run up to several millions of dollars. Such large investments are risky, given the fact that many SGH address small market niches with limited potential for return on investment. Therefore, any potential barriers to uptake, such as lack of credibility and evidence of effectiveness, compound the investment risk for developers.

### The Status Quo

To create theory-driven, evidence-based SGH, developers should collect and integrate scientific evidence and data throughout the entire development life cycle—from early stage theoretical work to later stage evaluation [1-3,5,7,15,16]. However, to date, there is no clear regulatory framework for the development of SGH beyond the type of evaluation data required (ie, evidence of risks and benefits). Regulatory requirements of SGH will likely depend upon their precise claims, and there are few transparent conditions that developers of minimal risk applications must meet before their products can be launched. This may also be the case for applications that are not obviously minimal risk as the developer must first engage regulatory authorities to determine what regulations they need to comply with.

In the absence of a regulatory framework, developers must look elsewhere for guidance on suitable approaches for developing SGH. Although a more robust level of evidence exists in the research literature, it is neither structured nor is there any clear consensus. The few resources that do exist are often focused on only a fragment of the development process, such as technology aspects or pedagogical aspects [17,18]. Others are described at such a high level that it is not possible for developers to implement such recommendations. Without clear consensus on frameworks, guidelines, and recommendations, developers must arbitrarily select which resources to follow.

This is in fact what happens. SGH developers currently use variable frameworks, differing guidelines, and alternative methodologies in SGH development [6,19]. The issue is further compounded by the fact that this emerging field is so multidisciplinary that each segment utilizes its own specific set of principles and frameworks to develop individual components. Moreover, development is often specialized to specific SGH classifications or target audiences [20,21]. Therefore, it is clear that SGH developers would benefit from the establishment of a defined set of requirements that represents the consensus views of SGH stakeholders [21]. Not only would this help increase SGH probability of success but it would also benefit the SGH community by raising the quality of SGH by providing the necessary evidence required by stakeholders. Moreover, it would also enhance the credibility of SGH developers and allow them to achieve a sustainable market share.

### Objective

The objective of our research was to search the literature and identify and evaluate the requirements, recommendations, and guidelines proposed by the SGH research community on the development of SGH. This included recommendations on what inputs are required to guide the development, what data should be collected, how games should be tested, which stakeholders should be engaged, and what game design approaches should be considered. On the basis of the findings, a clear and easy-to-implement consensus framework was developed to guide developers in the development of theory-driven, evidence-based SGH.

## Methods

### Databases and Search Strategy

A critical review of the research literature was performed in October to November 2018. The following databases were searched electronically: PubMed, ScienceDirect, Institute of Electrical and Electronics Engineers Xplore, CiteSeerX, and Google Scholar. A 3-step search strategy was used. An initial limited search was undertaken using the search strategy (game OR games) AND (serious OR applied OR health*), where * represents a wildcard to allow for alternative suffixes. This was followed by an analysis of the text words contained in the title and abstract and an analysis of the index terms used to describe article. A second search using all identified keywords and index terms was undertaken across all above databases. Finally, the reference list of all identified reports and articles were searched for additional relevant studies. A supplemental search of guidelines from regulatory authorities was also conducted to capture the requirements of these specific stakeholders. Three researchers independently evaluated the identified articles.

### Inclusion Criteria

Included papers were empirical research studies, literature reviews, opinion pieces, preliminary research, randomized controlled trials (RCTs), theoretical models, conference proceedings, conceptual frameworks, or design documents that (1) reported on the development or evaluation of a serious game for use in a health care context, (2) were published in English, (3) were published between January 2007 and November 2018, and (4) were peer reviewed.

### Exclusion Criteria

Excluded were any articles that (1) contained abstract only, (2) reported on serious games with applications outside of health care (formal education, corporate training, business decision making, etc), and (3) focused on pedagogical or psychological theory with no link to serious games.

### Coding

After screening, requirements relating to the development of SGH were extracted from each of the papers. These requirements were coded using the following steps, on the basis of a thematic analysis approach:

1. Requirements of SGHs that were described, identified, or documented within the included studies were collected verbatim.
2. Existing frameworks [2-5] within the included studies were evaluated to identify currently accepted terminology for various requirements. In cases of noncongruent terminology among frameworks, an established term was selected by 1 researcher and confirmed by the other 2 researchers.
3. The verbatim requirements from step 1 were reviewed individually, and duplicates (variations of the same underlying requirement) were removed. Again, the resulting term was selected by 1 researcher (CB) and reviewed and agreed by the other 2 researchers.
4. The requirements from step 3 were compared with the accepted terms from step 2 and, if deemed appropriate, the former were categorized within the latter. One researcher (CB) performed this step first and the coding was discussed and confirmed by the other 2 Researchers.

The coding was performed to facilitate understanding and to address parsimony, which is threatened by nonconsensus descriptions, terminology variations, etc.

## Results

### Search Results

**Figure 1:**
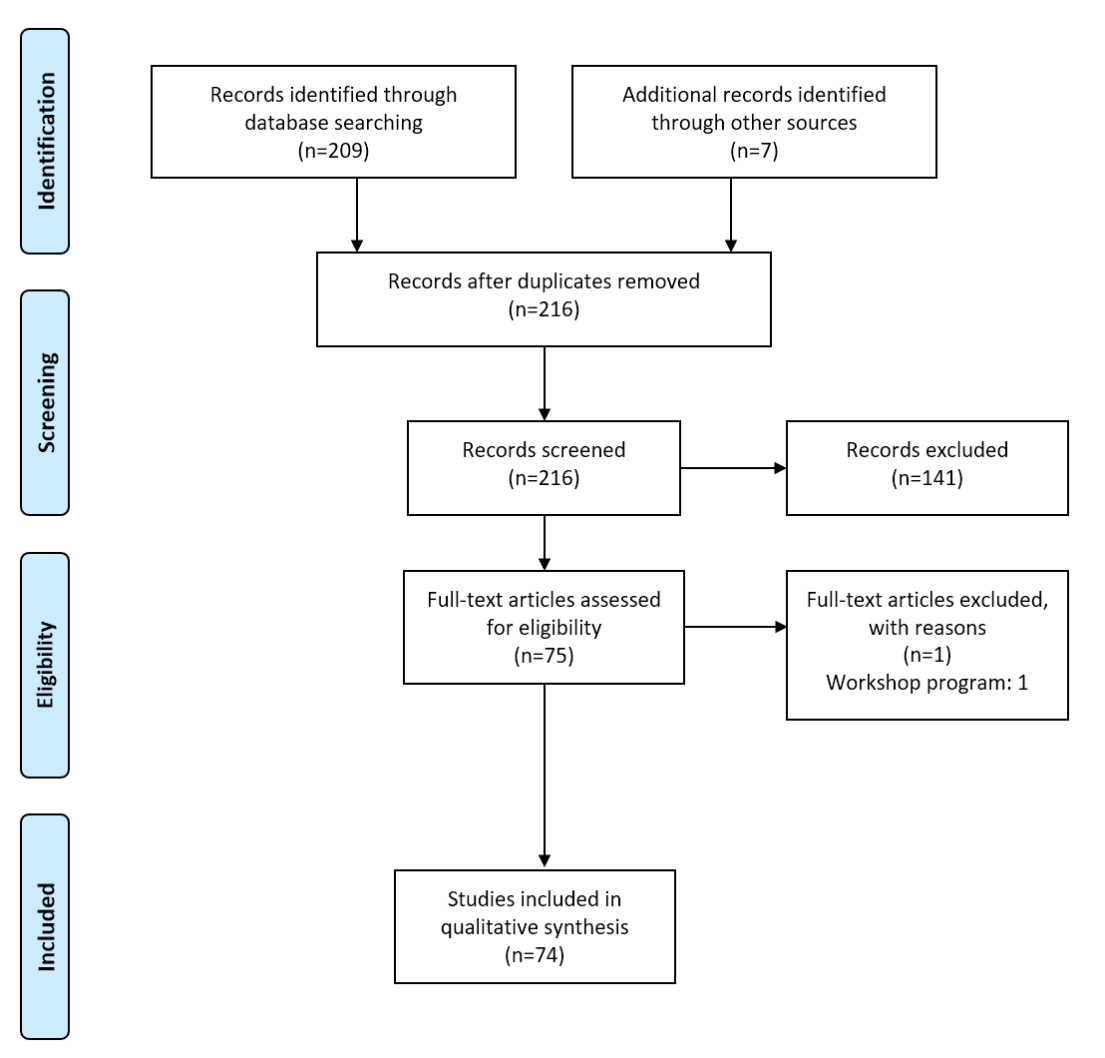
Literature search flowchart.

Our initial search yielded 216 papers (excluding duplicates). Of these, 74 papers were included in the review. See Figure 1 for a flowchart of the combined searches, as per Preferred Reporting Items for Systematic Reviews and Meta-Analyses guidelines [22]. Analysis resulted in a list of 62 requirements for the development of SGH, proposed by the SGH research community. Some requirements were formulated on a meta-level, whereas others were more detailed and concrete. The requirements were therefore categorized to allow for a structured analysis. We identified 5 categories of high-level requirements, as well as 20 detailed (low-level) requirements. See Table 1 for an overview of the identified requirements and categories, and the corresponding papers.

**Table 1:** List of high-level and low-level requirements and categories, and corresponding papers.

Hierarchy, category, requirements			References
**High level**			
	**Methodological approach**		
		Methodological approach	[2,3,5,7,16,23-28]
		Multidisciplinary, participatory design process	[1,2,5-7,12,13,19,21,23-25,29-37]
		Project management approach	[28,30,31,38-40]
		Quality evaluation	[1-3,12,15,16,19,23-26,28,32,35,36,38,41-54]
		Publish and disseminate findings	[1-3,16,24]
**Low level**			
	** Inputs **		
		Detailed profile of target audience	[1-3,5-7,23,33,43,45,51,55,56]
		Target audience needs	[2,5,7,16,25,29,43,55,57-60]
		Primary research goal	[1-3,12,15,23,25,33,43,55,58]
	**Models and theories**		
		Psychological theory	[1,2,4,23-25,27,53,56,58,59,61]
		Game mechanics	[1,2,5,7,19,20,33,44,45,47,54-58,62-66]
		Link between theories, mechanics, and game implementation	[7,19,21,23,26,44,45,55,58,62-64]
	**Game design**		
		Game type (genre)	[5-7,23,24,27,33,54,66,67]
		Game authoring tool	[68,69]
		Game platform	[5,6,33,67,70]
		Game engine	[2,19,31,71,72]
		Database	[2,19,43,70,72]
		Data protection	[3,2,7]
		Game objectives (explicit)	[1,3,6,19,20,23,33,47,48,53,54,56,65,73]
		Narrative	[6,7,20,21,23,27,31,40,43,48,53,56,58,62,65,73-76]
		Content	[6,7,19,23,36,54,65]
		Aesthetics and graphics	[7,19,20,23,27,29,40,47,49,53,56,67,73]
		Rules	[6,7,19,20,23,33,35,40,43,48,53,66,73]
		Challenge	[6,20,29,31,33,35,40,53,56,58,77]
		Interactions	[6,7,19,20,23,40,43,47,48,53,67,72,73]
		Feedback	[7,19,20,24,33,53,54,56,58,59,62,67,78,79]

### Evaluation Outcomes: High-Level Requirements

#### Methodological Approach

Out of 74 articles included for review, 11 articles stress the importance of employing an evidence-based, theoretically driven approach toward developing SGH. We identified both the research methodology and game design methodology requirements. The former includes the selection of clear outcome objectives at an early stage of development, as well as an evaluation of the game’s ability to achieve those objectives at a later stage. Without considering an overarching research methodology at the outset, game developers will be challenged, or will be unable, to evaluate their games with well-designed research studies. Kato identified the following 3 questions that ought to be answered by a research methodological approach: Who is your target audience? What is the primary research goal? How can the goals be reached through gameplay (relevant theories and models) [3]? The latter includes a structured approach toward profiling the target audience, assessing content and technical requirements, selection of relevant game mechanics, and the structured translation of outcome objectives and relevant theories and models into the game design.

#### Multidisciplinary, Participatory Design Process

Out of 74 articles, 21 articles advocate involving stakeholders from various disciplines in the development process. This is not surprising as SGH have emerged at the nexus of a wide variety of disciplines such as game design, software engineering, user experience design, health care, psychology, pedagogy, and clinical research. Various stakeholders were cited as relevant for inclusion in the design process, including research experts, clinical experts, regulatory authorities, and policy makers. Many acknowledge that a multidisciplinary approach poses a challenge, as individuals with differing backgrounds use differing terminology; it also highlights the importance of different elements and may be unaccustomed to working closely with those outside of their field. Nonetheless, this challenge is considered a necessary one. Importantly, 16 articles explicitly suggest also involving the target audience in the process.

A participatory or user-centered design process uses input and opinion from end users to inform a game developer’s choices. Although many acknowledge this as an important criterion for development, particularly in the field of mental health [37,79], there is currently no clear consensus on how the target audience should participate in game design or which elements it should inform. In fact, a recent meta-analysis on SGH with a behavioral focus indicated that certain types of participatory design may be more effective than others. Involving users in user testing and informant roles may be more beneficial than as co-designers, and the involvement in crucial aspects such as game dynamics elicited higher game effectiveness than involvement in trivial aspects such as esthetic components [29].

An iterative development approach was put forward by 6 articles. By developing SGH in segments, testing, and refining along the way, various stakeholders can inform at critical points of the development cycle, and development costs may be reduced.

#### Project Management Approach

Out of 74 articles, 7 articles highlighted the need for an approach that helps optimize the use of time and resources during development. This is particularly relevant for projects with limited development or research funding and SGH that focus on small market niches with limited potential for return on investment. There are particular challenges related to SGH that need to be managed: (1) helping multidisciplinary teams communicate and work together, (2) the slow process of research evaluation, and (3) the iterative prototyping process on the basis of user and expert feedback [30,38].

SGH developers increasingly use approaches inspired by Agile project management methods with SCRUM [31,39,80]. Agile is highly suited for complex projects where it is difficult to make a comprehensive implementation plan, and where many changes are expected along the way. Therefore, the approach focuses on conducting work in incremental iterations that can absorb new emerging insights and unanticipated changes. It is also based on the principle that multidisciplinary teams should self-manage their work and focuses on fostering communication and cooperation. However, there is currently no consensus on how to best adapt Agile approaches for participatory or user-centered design-focused projects [81,82] and how limits can be set on how far development iterations should go (how much feedback should be incorporated).

#### Quality Evaluation

Out of 74 articles, 30 articles cited the need to conduct quality evaluations and trials to validate SGH. This criterion is associated with the need to employ a high-level research methodology. The most important aspect of this criterion is likely the word quality, as there have been many trials of SGH, but only a few have reached a standard that can be considered high quality. Although it is evident that conducting a quality trial to validate SGH is a pivotal criterion, there is little consensus among the SGH community on what constitutes a quality trial. Many SGH trials only evaluate aspects, such as user experience, or technological aspects. Although this provides valuable information, it does not automatically allow for an assessment of how effective the game is at achieving the intended outcomes or the purpose for which it was designed. Drawing from the established research standards, a quality trial should include the use of a control group, participant randomization, an adequately powered trial, and objective measures of the primary and secondary outcomes.

Consistent with this thinking, several articles suggest that game developers should strive to carry out RCTs. Although these types of studies may not be necessary or relevant in all cases, RCTs are the still considered the gold standard for evaluating interventions in health care. Here, it is vital for game developers to work alongside stakeholders with expertise to determine the most relevant trial that will validate their games’ claims. The elements that need to be investigated are dependent upon the development stage of SGH. For example, at an earlier stage, SGH stakeholders suggest investigating usability, user experience, and duration of play.

For true evaluation, which typically occurs at a later stage of development, SGH stakeholders have identified the importance of evaluating a game’s efficacy (level to which it achieves intended objectives) in addition to its safety. The need for empirical evidence of efficacy and safety is consistent with requirements of health regulators, should SGH developers intend to have their product approved as a medical device.

Mixed-methods research, where both qualitative and quantitative data are gathered, is becoming an important methodology to investigate complex health-related topics. We identified several articles that highlight the need for better integration of these methods in the SGH research [41-43].

#### Publish and Disseminate Findings

SGH developers should endeavor to disseminate their findings to the SGH and wider health care community. This criterion was addressed in 5 articles. Consistent with other areas of health care where researchers are urged to publish all results, even negative ones, game developers should follow suit. This provides valuable evidence to the SGH community and may inform other researchers about what did and did not work for a target audience and game design. This is particularly important in assessing the effectiveness of serious games in relation to the constructs used in the design of the game.

### Evaluation Outcomes: Low-Level Requirements

In contrast to the high-level, or meta-level, requirements described above, the researchers also identified 20 low-level requirements from the SGH community. These requirements generally fell into 3 main categories: inputs, models and theories, and game design. Inputs represent the information and evidence that are integrated into the game from a conceptual perspective and cover the clinical or scientific content. SGH stakeholders clearly identified the importance of having a strong understanding of the target audience and their needs, clearly defining a research goal from the outset of a project. Models and theories were also identified as key requirements. This category represented the theories describing why a game would be expected to impact intended outcomes, and it also represented the associated link between these theories and game mechanics at the implementation level. SGH stakeholders suggest that without considering and integrating theories and models into SGH development, the resulting games are bound to be ineffective. The final and largest category of requirements contained all components related to the creation of the game itself. The category of game design comprised everything from defining one’s game authoring tool, engine, platform, and genre to the rules, challenges, and feedback that are integrated within the game. As identified in the models and theories categories, SGH stakeholders noted the importance of mapping the game inputs and model and theories to the game implementation choices and mechanics. Without this link, it is not possible to evaluate if the evidence and thinking captured in the former categories have been truly translated into the game.

### Proposed Framework

**Figure 2:**
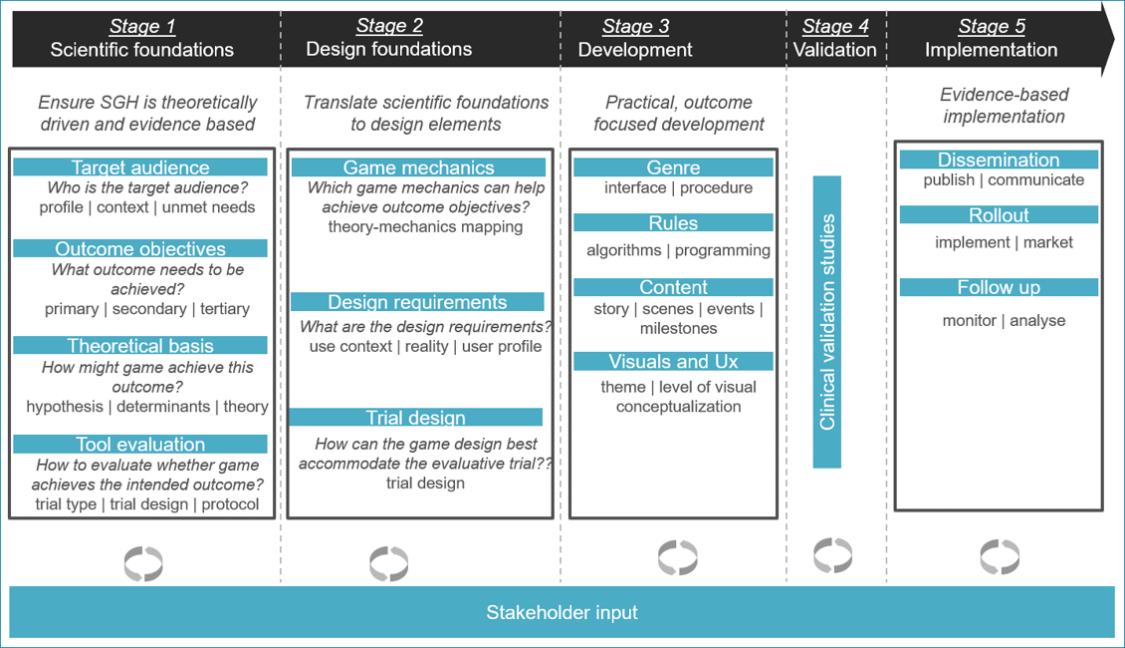
Proposed framework for developing serious games for health. SGH: serious games for health.

On the basis of the requirements suggested by the SGH research community, we propose a framework for developing SGH that comprises 5 distinct stages (Figure 2). Each stage has a specific focus and is informed by various stakeholders. Several iterations of development may occur within a given stage, progressively refining the SGH on the basis of testing with and feedback from relevant stakeholders. We will describe these stages as well as the stakeholder involvement in more detail below.

#### Stage 1: Scientific Foundations

Sound scientific foundations for the SGH should be established at the earliest stage of development. This will ensure that the final product is relevant, theoretically driven, and evidence based, in line with governing research methodological approaches. Although most developers tend to initiate the development process with a specific idea for an intervention in mind, the overarching objective of this stage is to assess at least conceptually and theoretically, on the basis of objective criteria, whether there is indeed a relevant medical unmet need for a clearly defined target audience who can be addressed with an SGH intervention. This stage typically comprises a topline review of the available literature on the target audience, disease status and impact, available treatment modalities, relevant clinical outcomes, psychosocial aspects (if any), and the governing health care landscape. To approach this task methodically, we propose that developers should focus on answering the following 4 questions: (1) Who is the target audience? (2) What outcome needs to be achieved? (3) How might SGH achieve this outcome? and (4) How can we evaluate whether SGH achieve the intended outcome?

##### Who Is the Target Audience?

A first, limited profile of the intended end users should be constructed. Information can be obtained through literature review, explorations of Web materials such as patient fora and websites, or consultation with subject matter experts (eg, medical specialists and patient organizations). At this stage, the profile should at minimum cover who the target audience is, the context in which the people function, the specific problems they face, and what their unmet needs are (eg, what alternatives are available to them and how well do these other interventions address those needs?). On top of this, some details may be required that are game- or topic-specific. For example, if the game intends to help children with disabilities improve their motor skills, it is important to understand the disease status and specific medical needs. For a game aimed at helping patients with schizophrenia reintegrate in society, the profile should also include an overview of the other stakeholders who play a role in these patients’ lives and the relations among them (psychosocial details). At the other end of the spectrum, a game intended to educate medical professionals on how to handle ethical dilemmas would require some level of insight into the context in which these professionals make decisions, their learner profiles, and pedagogical needs. In stage 2 of the development, this first limited profile will be broadened to also include information regarding the specific game design needs of the target audience (eg, user experience needs and usability needs).

##### What Outcome Needs to Be Achieved?

Outcome objectives should be clearly formulated before any creative or technical development starts. These outcomes should be (medically) relevant, based on objective criteria. Due to the nature of SGH and their ability to educate, empower, and address multiple domains of health, we propose a biopsychosocial approach toward identifying relevant outcome objectives [83]. This model considers not only the biological factors of human functioning in the context of health but also the psychological and social factors. An often-cited example of where this is particularly relevant is the issue of therapy compliance. A majority of patients do not comply with the treatment regimens that could save their lives [84]. The solution to compliance issues is clearly complex, and psychological and behavioral factors play a prominent role. This requires developers to evaluate the biological, psychological, and social context of a disease or health condition [85].

Although outcome objectives may range from clinical to pedagogical, psychological, or behavioral, it is important to primarily identify a single primary outcome objective. This will steer the subsequent steps of game development and provide the greatest opportunity for success. This does not exclude a developer from identifying secondary (or even tertiary) outcome objectives as well. In fact, many cases will require the identification of various secondary outcomes that are closely linked to the primary outcome, and these need to be incorporated in the game construct as well. To give a few examples, if improved therapy compliance is the primary outcome objective, secondary outcomes may be to improve the quality of the patient’s relationships or provide a more stable home environment (eg, for psychiatric disorders), or the secondary outcomes may be to overcome the patient’s misconceptions or erroneous beliefs about the therapy (eg, for chronic patients fearing dependence on long-term medication) [86,87] or to make physical rehabilitation exercises more fun and rewarding (eg, for kids with motor skill disorders).

##### How Might Serious Games for Health Achieve This Outcome?

An often-overlooked step in SGH development is to formulate a hypothesis of how a game might achieve the intended outcome objective(s). Formulating such a hypothesis is a vital step toward the purposeful design of SGH and the evaluation of their causal effect on the outcome. First, developers should identify the outcome determinants. Outcome determinants are the underlying factors or parameters that directly or indirectly determine or influence the outcome objective. For example, if the primary outcome is to reduce peri-operative pain in children and the secondary outcome objectives are to reduce these children’s peri-operative anxiety and stress (which are closely related to the primary outcome objective), it is important to establish what underlying factors contribute to pain, anxiety, and stress in children in this situation and evaluate which of these factors a game might positively or negatively impact, and how. The literature reveals that pain has sensory, emotional, cognitive, and behavioral components that are interrelated with environmental, developmental, sociocultural, and contextual factors [88]. One determinant is the level of pain medication administered to the child after surgery, with inadequate levels resulting in more pain. Delving deeper into the research literature reveals that parents play a key role in managing their child’s pain in the home setting postsurgery. Any misconceptions parents have about pain medication may result in inadequate levels of pain relief, thereby increasing the child’s pain experience. In this case, SGH may leverage pedagogical models and approaches toward educating parents about pain relief and correcting erroneous beliefs. Another determinant of peri-operative pain, anxiety, and stress is lack of control. Developers should therefore investigate whether SGH may help increase a child’s feeling of control of the situation, for instance, through teaching coping skills or by allowing them to freely explore the peri-operative setting and events associated with it, so that they can anticipate what lies ahead.

The identification of these determinants and relevant underlying models or theories should occur in consultation with experts from relevant disciplines. From a biopsychosocial perspective, the link between psychosocial determinants and clinical outcome also needs to be understood and integrated in the game design. In addition, the role of the target audience as well as all relevant stakeholders needs to be evaluated. As such, this step of the process is one of identifying the various factors that contribute to the problem, grouping them, charting how they relate to and impact one another, and establishing hierarchies and relative weights of impact and importance. These insights will inform the game construct, narrative scenarios, and mathematical algorithms further on in the development process.

##### How Can We Evaluate Whether Serious Games for Health Achieve the Intended Outcome?

Before development starts, developers need to think ahead of how they plan to evaluate whether the game achieves the intended outcome(s). In 2012, Kato first formulated guidelines for conducting high-quality evaluations of SGH [1]. These suggest conducting randomized (clinical) trials that include adequate numbers of participants as well as control groups, the use of objective outcome measures alongside self-reports, monitoring and reporting potential negative side effects, and consulting research experts early on to guide the design of quality trials (eg, measures, n numbers, statistical power, and trial length).

Game evaluation should also include an evaluation of broader intervention characteristics, such as perceived relevance, user experience, and user friendliness. The particular characteristics that need to be evaluated vary depending on the objectives, and they may include satisfaction of needs (competence, autonomy, and relatedness), ability to engage, level of motivation, and competence autonomy. Ideally, a mixed-method methodology is used, in which both quantitative (eg, surveys) and qualitative (eg, focus groups and interviews) data are collected for analysis and evaluation.

This evaluation can be done throughout the development process through a series of iterative tests with the target audience and other stakeholders, using standard measures such as Intrinsic Motivation Inventory and Player Experience of Need Satisfaction Scale [89,90].

#### Stage 2: Design Foundations

Game developers can draw from a wide range of game mechanics, design, and technological features to construct SGH. If the SGH are to achieve the intended outcomes, the choice of these game mechanics, design, and technological features should be guided by the scientific foundations established in stage 1. It is imperative to translate the theoretical basis into relevant, implementable game design elements. This stage therefore aims to answer the following 3 questions: (1) Which game mechanics are best suited to achieve the intended outcome objectives? (2) What are the design requirements? and (3) How can the game design best accommodate the evaluative trial?

##### Which Game Mechanics Are Best Suited to Achieve the Intended Outcome Objectives?

Game mechanics are rules or methods that define the interactions and flow of a game session. They describe interactions, game conditions, and triggers in an abstract manner. The most frequently employed game mechanics in electronic health are currently rewards and feedback [44], but other examples include turn-taking, story, penalties, realism, and protégé effect. In the past, game developers often decided upon a game genre before selecting what game mechanics to use. However, as game mechanics are more instrumental toward achieving the intended outcome objectives, developers should first work together with relevant subject matter experts to map the outcome objectives, models, and theories identified in stage 1 onto relevant game mechanics before settling on a particular game genre [44]. In mapping the scientific foundations onto these mechanics, developers gain insight into which game mechanics should be used to effectively achieve the intended outcomes. Although this is a relatively novel approach, there are currently several well-documented examples in the research literature of how this can be done for SGH that have a pedagogical or behavioral focus [45,62,63]. These types of SGH often have outcome objectives that pertain to either *understanding* or the acquisition of a specific skill set (eg, communication skills and coping skills). Depending on the type of outcome envisaged, there may be layers of intermediate learning objectives that need to be addressed. Here, the pedagogical or behavioral intents should be mapped to a low-level game mechanic implementation. In 2015, Arnab proposed a model for translating learning objectives into learning mechanics and mapping these to relevant game mechanics [45]. This so-called learning mechanics-game mechanics model guides developers in the development of more effective, pedagogy-driven SGH, as it ensures that game mechanics are chosen on the basis of their ability to contribute toward the intended outcomes. In the example of reducing peri-operative pain, stress, and anxiety in children, one such learning objective may include remembering the sequence of events for the upcoming procedure (knowing what to expect and do). This involves the thinking skills *understanding* and *retention*. Several learning mechanics address these thinking skills: *exploration*, *repetition*, and *planning*. Each of these learning mechanics can in turn be mapped onto one or more game mechanics, for example, *story*, *cascading information*, and *strategy and planning*. As such, the scientific foundations established in stage 1 can be translated into the game construct.

##### What Are the Design Requirements?

At this point, the target audience profile needs to be broadened to guide the design choices. Although the specifics will depend on the objective and scope of SGH, the objective is to gain insight into (1) the context of use and (2) the reality of the target audience. What context will the tool be used in? Will there be access to special equipment or technical support? Will the tool be used at home or in hospital? How realistic does the tool need to be (level of fidelity and immersion)? If it needs to be realistic, what characters does the target audience meet or interact with? What type of environments do they move about in? What situations or dilemmas do they typically encounter? In addition, information regarding optimal user experience for the target audience should be collected. This includes computer literacy skill levels, literacy and numeracy levels, and possible physical or mental limitations that may pose restrictions on game design (eg, epilepsy, auditory problems, and limited motor function). This type of information can be gathered through interviews, time-and-motion exercises (shadowing a typical user for a day), or focus groups with the target audience or relevant experts.

##### How Can the Game Design Best Accommodate the Evaluative Trial?

Are there any design considerations with respect to the future evaluation of the SGH? For instance, if data need to be collected, should this data collection be included in the game design? (eg, tracking user response time, motion ranges) Will it be collected out with the game format (eg, pre and postgame interviews, clinical scales, and biologic sampling). Does it require live feedback or investigator intervention during game play? Are there any design considerations for use in a clinical environment? Should the game design include components that can help track or assess user experience (eg, level of immersive play, eye tracking, etc)?

#### Stage 3: Game Development

Once stage 2 has been completed, developers should have sufficient scientifically grounded input to guide the practical development of the game. Various approaches can be used depending on the complexity, the developer’s resources, and software and technological skills, but overall, the process comprises the selection and development of the (1) game genre, (2) game rules, (3) content, and (4) visuals and user interface. This stage ideally occurs in an iterative, participatory manner, involving key stakeholders such as clinical experts and target users to informally test and refine the tool along the way.

##### Game Genre

The scientific and design foundations developed in stages 1 and 2 should now enable developers to select the most appropriate interface genre (eg, first person, third person, and isometric) and procedure genre (eg, strategy game, adventure game, and shooter game) for the intended target audience and context of use. The genre chosen should facilitate the incorporation of the game mechanics and design requirements identified under stage 2.

##### Game Authoring Tool

There are several authoring tools available for game development, and it is important to assess upfront which authoring tool is most suitable for the project. This will largely depend not only on the technical capabilities available in the team (eg, team members with specific coding skills) but also on whether future administrators of the SGH will need to make their own modifications, such as adding new narratives or including new data measurements. Considerations should be given to open-source authoring tools versus licensed authoring platforms.

##### Game Rules

Developers can now draw up a set of game rules that specifies how the player’s actions impact the game environment. Depending on the intended purpose of the tool, these rules may need to be consistent and transparent (to allow players to strategize on the basis of their knowledge of the rules) or hidden or unpredictable (to force players to truly reflect on their choices rather than making decisions that help raise game scores). Such rules are often described in mathematical algorithms that govern the tool’s programming. When the SGH need to closely reflect reality to achieve the intended outcome objectives, for instance, through use of realistic narratives or life-like responses to in-game decision making, developers will need to translate the relations, hierarchies, impacts, and relative weights of importance of the outcome determinants, identified in stage 1, into a mathematical algorithm. This will facilitate procedurally generated narrative branching and can drive feedback and reward approaches. To stimulate flow and user engagement, developers can also build in rules that adapt game difficulty and other game-play elements to the performance or physical or mental state of the user.

##### Game Content

The amount of content required in a game will vary substantially depending on the intended objectives. Many SGH require at least some instructional content or a narrative that ties everything together. When SGH have a large pedagogical or behavioral focus, the narratives can become more elaborate, ranging from linear stories to nonlinear stories that have branching and even offer multiple endings. Within the context of health, narratives are a valuable resource to generate an understanding of the impact of an illness on the patient’s life and well-being [91,92]. Narratives are an everyday medium that people use to communicate information to one another, and therefore they are a familiar format to users [93]. Narratives are perceived as providing essential emotional and social information not usually found within routine resources that lend meaning and perspective to a patient’s predicament [94].

Developers should develop the game content in function of the intended outcomes. Linear story lines may be less time consuming, but they also tend to reduce the potential efficacy of the narrative as a persuasive mechanism as it is not responsive to the users, their state, or in-game decisions. Many SGH are designed using a one-size-fits-all approach; however, recent research shows that this approach may not be effective as different types of people are motivated by different persuasive strategies [95], and a strategy that worked well with one group of people may actually demotivate a different group [95,96]. Personalization has also been shown to be important for successful impact [97]. The relevance of SGH is often directly related to their ability to capture the patient’s unique reality and circumstances in the content [93,98]. In addition, features that allow patients to self-personalize content may promote autonomy and empower patients to take ownership over health care decisions [99]. Developers should also address (health) literacy and numeracy profiles of the intended target audience to maximize chances of success [100-104]. Therefore, building on the target audience profile and design requirements identified in stage 2 allows developers to take a more informed approach toward game content.

##### Game Visuals and User Interface

On the basis of the specific target audience and design requirements, a theme needs to be chosen, which specifies the overall look and feel of the tool (colors, sounds, environments, characters, navigation, interface, etc). At the same time, developers will need to assess what level of visual conceptualization will be needed. In some cases, the use of archetypical symbols or icons may be warranted to convey complex concepts either to avoid information overload or to eliminate bias [101,105,106]. Graphics have also shown to impact the emotional response of participants [107].

#### Stage 4: Game Evaluation

The game has now been developed, informally tested, and refined with users, and it should be ready for (clinical) evaluation. Once the trial sites and investigators have been chosen, ethical committee or other approvals have been granted, and users have been recruited for the evaluation study, developers can commence the evaluation, analysis, and assessment of whether the tool successfully achieves the intended outcomes. This stage ideally occurs in consultation with relevant research experts who can guide and oversee the evaluation studies and support the analysis of collected data.

#### Stage 5: Implementation

On the basis of the findings of stage 4, developers may wish to further refine and reevaluate updated versions of the tool or proceed immediately with rollout toward the intended target audience. Regardless of the outcomes of the game evaluation studies, developers should try to disseminate the study findings to the wider SGH research community, as this will help further advance the field. Even publishing and communicating null results may provide insight on how to optimize SGH interventions and provide guidance on the best practices and pitfalls to avoid. If a game is successfully validated and implemented or marketed for the intended target audience, efforts should be made to collect user data in the field, to help monitor for adverse events (if relevant), or to further explore the validity and use of the SGH.

#### Stakeholders

More research should be done to identify which stakeholders should be involved in the design process, to understand how to engage them, and at what stage of the development. We propose to consider at least the following 4 stakeholders.

##### Subject Matter Expert

At the earliest stage of development, subject matter experts are well placed to provide input and guidance on the 4 questions that should be answered when establishing the scientific foundations.

##### Target Audience

When deciding on the level of user involvement, one should balance the need for input from users with the availability of resources such as time and funding [32]. One should only involve end users at relevant stages of development. We propose to engage them at specific points in stages 2, 3, and 4, when their input and participation are most likely to yield valuable, accurate information and feedback. At stage 2, end users should be engaged to broaden the profile research and identify their specific design requirements that are crucial to tool effectiveness (ie, not esthetic design perspectives). The purpose of the contact is to determine in more detail who the end users are, which subgroups exist, what their socioeconomic backgrounds are, their day-to-day reality, and other aspects that may inform game design, such as literacy levels, numeracy levels, computer skills, and understanding of subject matter. Many established formats to engage end users are available to developers. Examples include time and motion exercises in which developers shadow end users during a typical day in their life or during a relevant event, such as a hospitalization.

At stage 3, end users should be actively engaged in the testing of early prototypes of the game to gather feedback on such aspects as user experience, content relevance, realism, graphic design features (minimal), and preliminary assessment of the achievability of outcome objectives. It is important to note here that not all user feedback needs to be incorporated. To avoid *feature creep* and ensure effective use of project resources, subject matter experts can help evaluate which feedback should be prioritized. Ideally, an emphasis is placed on feedback that will help enhance user uptake and SGH effectiveness.

At stage 4, end users should be recruited into a quality trial to validate the game. End users who participate in trials should not be involved in the earlier stages of the development.

##### (Clinical) Research Expert

Research experts should be engaged to advise on scientific approach and trial design at an early stage of development and ideally during the trial.

##### Business Expert

To ensure market readiness and effectively implement and roll out the SGH to the market, a business expert should be consulted, ideally no later than stage 3.

Aside from these 4 stakeholders, it may be relevant to consult with regulators, health care professionals, patient organizations, health technology assessments, and others throughout the various stages of the development process.

Working with multiple stakeholders carries a specific set of challenges such as clear alignment on roles and responsibilities, finding a common language, understanding of limitations, and consensus on priorities and outcomes [30,55]. These may be overcome, at least in part by (1) educating stakeholders about the development process and the tools and methods used, by means of interactive, hands-on workshops, (2) forming an advisory board of key stakeholders which meets on a regular basis to discuss the project, and (3) by assigning a project manager who functions as the go-to person for all stakeholders involved and who can make final judgment calls in case of conflict, in line with the project’s intended objectives and within the stipulated resource limitations.

## Discussion

A review of existing literature, recommendations, and guidelines on SGH development has allowed us to formulate a framework for developing theory-driven, evidence-based SGH that represents many of the requirements set out by SGH stakeholders in the research literature. The framework covers all aspects of the development process (scientific, technological, and design) and is transparently described in sufficient detail to allow developers to implement it in a wide variety of projects, irrespective of discipline, health care segments, or focus. Adoption of such a consensus framework by the wider SGH research community is a first step toward increasing probability of success and raising the quality of SGH by providing the necessary evidence required by stakeholders. Moreover, it would also enhance the credibility of SGH developers and allow them to achieve a sustainable market share.

## Abbreviations

RCT: randomized controlled trial
SGH: serious games for health
