# Correction: Developing Theory-Driven, Evidence-Based Serious Games for Health: Framework Based on Research Community Insights

**DOI:** 10.2196/18515

**Published:** 2020-04-28

**Authors:** Sarah Verschueren, Connor Buffel, Geert Vander Stichele

**Affiliations:** 1 MindBytes BVBA Merksplas Belgium; 2 MindLab Interactive AI Inc Edmonton, AB Canada

In “Developing Theory-Driven, Evidence-Based Serious Games for Health: Framework Based on Research Community Insights” (JMIR Serious Games 2019;7(2):e11565), an error was found in Reference 2 of the reference list. Arnab S was listed as the author, but should have been listed as the lead editor, instead. The correct author of the book is Kato PM, instead of Arnab S. The correct reference is:

Kato PM. The role of the researcher in making serious games for health. In: Arnab S, Debattista K, Dunwell I, editors. Serious Games for Healthcare: Applications and Implications. Hershey, Pennsylvania: IGI Global; 2013:213-231.

This correction will appear in the online version of the paper on the JMIR website on April 28, together with the publication of this correction notice. Because this was made after submission to PubMed, PubMed Central, and other full-text repositories, the corrected article has also been resubmitted to those repositories.

